# Crystal Structure Prediction of Binary Alloys via Deep Potential

**DOI:** 10.3389/fchem.2020.589795

**Published:** 2020-11-26

**Authors:** Haidi Wang, Yuzhi Zhang, Linfeng Zhang, Han Wang

**Affiliations:** ^1^School of Electronic Science and Applied Physics, Hefei University of Technology, Hefei, China; ^2^Yuanpei College of Peking University, Beijing, China; ^3^Beijing Institute of Big Data Research, Peking University, Beijing, China; ^4^Program in Applied and Computational Mathematics, Princeton University, Princeton, NJ, United States; ^5^Laboratory of Computational Physics, Institute of Applied Physics and Computational Mathematics, Beijing, China

**Keywords:** many-body potential energy, deep learning, crystal structure prediction, Al-Mg, alloy

## Abstract

Predicting crystal structure has been a challenging problem in physics and materials science for a long time. A reliable energy calculation engine combined with an efficient global search algorithm, such as particle swarm optimization algorithm or genetic algorithm, is needed to conduct crystal structure prediction. In recent years, machine learning-based interatomic potential energy surface models have been proposed, potentially allowing us to perform crystal structure prediction for systems with the accuracy of density functional theory (DFT) and the speed of empirical force fields. In this paper, we employ a previously developed Deep Potential model to predict the intermetallic compound of the aluminum–magnesium system, and find six meta-stable phases with negative or nearly zero formation energy. In particular, Mg_12_Al_8_ shows excellent ductility and Mg_5_Al_27_ has a high Young's modulus. Based on our benchmark results, we propose a relatively robust structure screening criterion that selects potentially stable structures from the Deep Potential-based convex hull and performs DFT refinement. By using this criterion, the computational cost needed to construct the convex hull with *ab initio* accuracy can be dramatically reduced.

## Introduction

In recent years, crystal structure prediction has played an increasingly important role, not only for understanding the ground-state structure of matter, but also for designing materials and drug molecules with desired functionality (Oganov, 2018; He et al., 2019; Zhao et al., 2019; Xie et al., 2020). Generally speaking, a ground-state crystal structure prediction method involves three components: a model that generates the interatomic potential energy surface (PES) and forces, a sampling technique for exploring different conformations in the configuration space, and a relaxation procedure to find the local minima on the PES (Podryabinkin et al., 2019). While the relaxation procedure is relatively standard, different sampling techniques have been championed by different software packages. For instance, the genetic evolutionary algorithm, the particle swarm algorithm, and the firefly algorithm have been widely used in the USPEX software (Glass et al., 2006), the CALYPSO package (Wang et al., 2010), and the PyChemia library (Avendaño-Franco and Romero, 2016), respectively. Due to the high accuracy required by both sampling and relaxation, density functional theory (DFT) (Kohn and Sham, 1965) is typically used for generating the PES. Despite its widespread success, DFT has a high computational cost that typically scales cubically with the system size, which, to some extent, hinders routine applications to large and complex systems.

Many empirical PES models for popular solid-state systems have been proposed (Mendelev et al., 2009; Jelinek et al., 2012; Dickel et al., 2018) to address the efficiency issue of DFT. Due to the relatively simple and analytical expressions adopted by these empirical models, an acceleration of many orders of magnitude in terms of computational cost can be gained, but presumably at the price of accuracy and transferability. As such, trail-and-error processes are typically required for developing such models, yet challenges have remained for systems involving multiple elements, complex and exotic phases, or bond breaking and formation events.

In recent years, a few machine learning (ML) techniques have been proposed for representing the PES (Behler and Parrinello, 2007; Bartók et al., 2010; Artrith and Urban, 2016; Khorshidi and Peterson, 2016; Shapeev, 2016; Han et al., 2018; Zhang et al., 2018a,b). Unlike typically empirical PES models, representations coming from ML tasks, such as kernel functions and neural networks (NNs), have shown great promise to fit high-dimensional functions. When trained on a suitably generated dataset of atomic configurations and corresponding potential energies and forces, a good ML-based PES model can be used with an accuracy of the reference DFT model, and an efficiency comparable to that of empirical PES models. Not surprisingly, ML-based PES models have been employed in recent work for structure search tasks. For instance, boron has been studied by several groups: Podryabinkin et al. (2019) adopted the moment tensor potential (Shapeev, 2016) and the USPEX evolutionary algorithm; Huang et al. (2018) used the Behler–Parrinello potential (Behler and Parrinello, 2007) and the stochastic surface walking global optimization method; Tong et al. (2018) used the Gaussian Approximation Potential (Bartók et al., 2010) and the CALYPSO approach.

In this work, we target at using ML-based PES models for crystal structure prediction of alloys. We adopt the smooth version of the Deep Potential (DP) model (Zhang et al., 2018b), which employs NN architectures to parameterize two networks, the embedding network that defines a list of symmetry-preserving descriptors, and the fitting network that maps these descriptors to local energy contributions. The versatile architecture of DP makes it particularly suitable for multicomponent systems and those involving bond breaking and formation, for which most methodologies are hard to handle. The aluminum–magnesium (Al–Mg) binary alloy system is selected as an example based on the following reasons: First, Al–Mg binary alloys are important in real-life applications. They are widely used in automotive, aerospace, and electronic device industries (Gupta and Ling, 2011) due to their lightweight nature and excellent mechanical properties. However, only a limited number of intermetallic compounds of the Al–Mg binary system have been documented in well-known databases, such as the American Society for Metals (ASM) Alloy Phase Diagram Database^1^, the Inorganic Crystal Structure Database (ICSD)^2^, the Open Quantum Materials Database (OQMD)^3^, and the Material Project database (MP)^4^. Second, our previous study has established an Al–Mg DP model (Zhang et al., 2019), which describes well the basic physicochemical properties and has been carefully tested. As such, this DP model can be readily used for crystal structure prediction and can be download online^5^.

Combining the particle swarm optimization (PSO) method and the DP model, potential intermetallic compounds of the Al–Mg system are systematically explored. Compared with a previous study (Zhuang et al., 2017), which only explored the Mg-rich phases, our simulation covers a much wider concentration range. Six new Al–Mg intermetallic compounds (Mg_12_Al_8_, Mg_7_Al_9_, Mg_14_Al_18_, Mg_6_Al_10_, Mg_8_Al_16_, and Mg_5_Al_27_) are found to be meta-stable. The mechanical properties of these new compounds are further investigated. To facilitate future investigations of more complicated tasks, special attention is given to the whole simulation protocol and the selection criterion for further DFT validations. Direct comparisons with popular empirical PES models and DFT show the advantage of DP in terms of both accuracy and efficiency.

## Computational Methods

We adopt the PSO method, as implemented in the CALYPSO package (Wang et al., 2010), to search potentially stable and meta-stable Mg–Al intermetallic structures. PSO is inspired by the choreography of a bird flock and can be seen as a distributed behavior algorithm that performs multidimensional search. In the CALYPSO package, there are three steps for a global structure prediction task. First, a group of structures called population is generated randomly with symmetric constraints to allow a diverse sampling of the PES. The number of structures employed here is defined by a parameter called population size (PopSize). Second, a local relaxation of the population is performed based on a PES engine, which is typically a DFT model, and here we replace it with a DP model. A procedure that eliminates similar structures by using the so-called bond characterization matrix is followed up to enhance the search efficiency. Third, a certain number of new structures (the best 60% of the population size) are generated by PSO. Within the PSO scheme, a velocity vector associated with each structure is updated using the information of the previously proposed and optimized structure, as well as the globally best structure, that is, the structure with the lowest enthalpy, at the current generation. The new structures are generated based on the current structures and the velocity vectors. The last two steps continue iteratively until the predefined largest number of generations (GenNumb) is reached. The parameter GenNumb is typically selected to be large enough so that the structure with the lowest energy can sustain for several generations. Generally speaking, the more atoms (Natom) in a structure, the larger PopSize and GenNumb are required. We refer to Yanchao Wang and Ma (2012) for more details of the CALYPSO code. We set the CALYPSO parameters according to the following criteria:

(1) $$\begin{array}{l} \{\begin{array}{lllll} \text{PopSize} & =30, & \text{GenNumb} & =60; & \text{if }\text{Natom}\leq 10,\\ \text{PopSize} & =40, & \text{GenNumb} & =80; & \text{if }10<\text{Natom}\leq 20,\\ \text{PopSize} & =50, & \text{GenNumb} & =100; & \text{if }20<\text{Natom}\leq 32. \end{array} \end{array}$$

The DP model (Zhang et al., 2018b) used here employs NN functions to represent the PES. In short, the total energy of a system is described as the sum of atomic energies,

(2) $$\begin{array}{l} E=\overset{N}{\underset{n=1}{\sum }}\epsilon_{i}, \end{array}$$

where ϵ_*i*_ is the *i*th atomic energy. The atomic energy is represented as

(3) $$\begin{array}{l} \epsilon_{i}=N_{\omega \left(i\right)}\left({\left\{R_{ij}\right\}}_{j\in N\left(i\right)}\right), \end{array}$$

where *N*_ω(*i*)_ is called a sub-network that computes the atomic contribution to the total energy, and ω(*i*), which depends on the chemical species of atom *i*, denotes the weights used to parameterize the sub-network. The neighbors of atom *i* within the cut-off radius *R*_*c*_ are denoted by N(i). *R*_*ij*_ is the position of atom *j* relative to *i* used to describe the local environment of atom *i*. To generate uniformly accurate DP models in a way that minimizes both human intervention and the computational cost for data generation and model training, a concurrent learning strategy called the Deep Potential GENerator (DP-GEN) (Zhang et al., 2019) has been proposed. In this strategy, an initial dataset (random Al–Mg alloy structures) labeled by DFT calculations is used to train an ensemble of DP models, and molecular dynamics is driven by one of the DP models to sample the configuration space. An error indicator serves to select a small fraction out of the new samples as candidates, which are labeled with *ab initio* energies and forces and added to the database. Such iterations are repeated until the configuration space has been explored sufficiently, and a decent DP model has been obtained with high accuracy and transferability. The training is performed using the DeePMD-kit package (Wang et al., 2018) and the concurrent learning strategy is realized by the DP-GEN software package (Zhang et al., 2020). In details, the DeepPot-SE model is used with a cutoff radius of 8.0 Å. The size of the embedding and fitting NNs are 25 × 50 × 100 and 240 × 240 × 240, respectively. During the training, the learning rate decreases exponentially with respect to the starting value of 0.0005. The decay rate and decay step are set to 0.95 and 128,000, respectively. In addition, the prefactors of loss functions are set to $p_{e}^{start}=0.02,p_{e}^{limit}=2,p_{f}^{start}=1000$, $p_{f}^{limit}=1,p_{v}^{start}=0.0,p_{v}^{limit}=0.0$. Both DeePMD-kit and DP-GEN are publicly available online^6^. For more details, we refer the reader to Wang et al. (2018) and Zhang et al. (2019, 2020).

All DFT calculations are carried out with the Vienna Ab-Initio Simulation Package (VASP, version 5.4.4) (Kresse and Furthmüller, 1996). The generalized gradient approximation within the Perdew–Burke–Ernzerhof (Perdew et al., 1996) (PBE) functional is used to model the exchange-correlation energy. The plane wave basis sets with kinetic energy cutoff of 600 eV are used to expand the valence electron wave functions. For all structural relaxations, the convergence criterion for the energy in electronic SCF iterations and the Hellmann–Feynman forces in ionic step iterations are set to 1.0 × 10^−6^ eV and 1.0 × 10^−2^ eV/Å, respectively. The Brillouin zone is represented by Monkhorst–Pack (Pack and Monkhorst, 1977) special k-point mesh with a grid spacing of 0.08 Å^−1^. The phonon spectra are obtained based on finite-difference method as implemented in the Atomic Simulation Environment (ASE) (Bahn and Jacobsen, 2002; Larsen et al., 2017) software, where the forces are calculated by the python interface of DeePMD-kit. To calculate the phonon density of states, the q-point mesh is set to 20 × 20 × 20. The local structure relaxation is carried out by the LAMMPS package (Plimpton, 1995), and the DP model used here has been reported and extensively tested in Zhang et al. (2019).

All structure data and convex hulls are analyzed by pymatgen software (Ong et al., 2013).

## Results and Discussions

**Figure 1:**
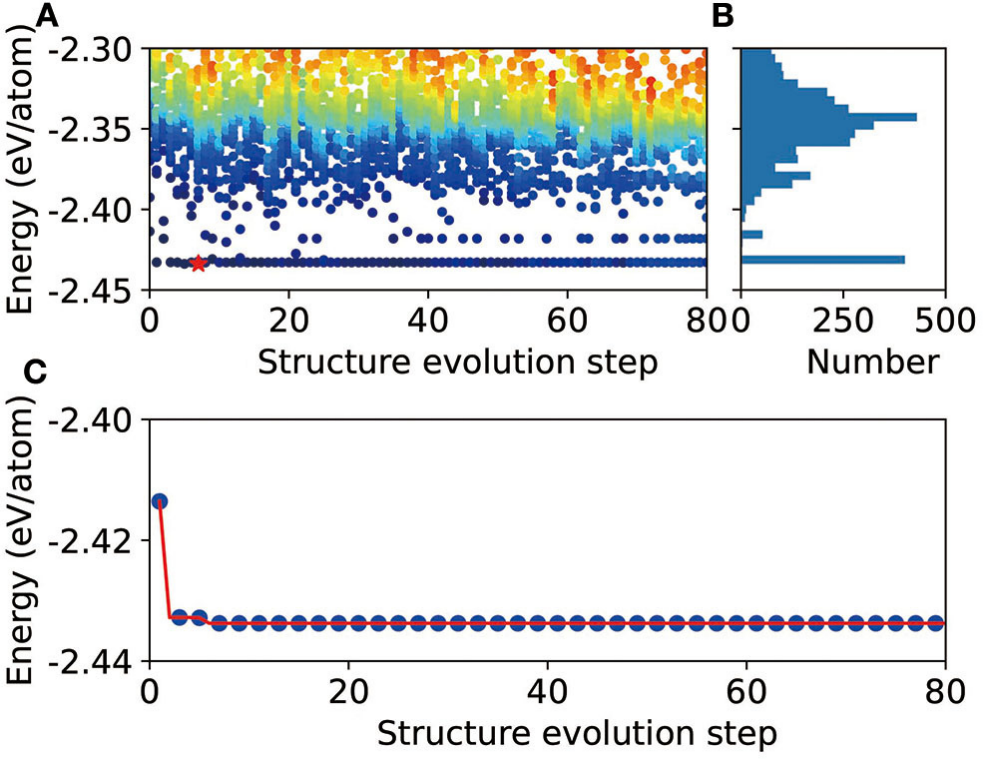
Schematic illustration of the DP+CALYPSO process for the Mg_12_Al_8_ system. **(A)** Evolution of the energies of all structures during the structure prediction process. The red star indicates the global minimal structure. **(B)** Energy histogram. **(C)** Evolution of the lowest energy during the structure prediction process.

To demonstrate the validity of CALYPSO+DP scheme, we perform some preliminary tests for several different stoichiometric proportions. Here, we take Mg_12_Al_8_ as an example to show the evolution of the energies of all the structures (Figure 1A), as well as the lowest energy (Figure 1C), during the CALYPSO structure search process. According to the energy histogram in Figure 1B, it can be found that there are about 458 structures, out of a total number of 3,200 structures, within an energy range <20 meV/atom (compared with the ground state structure). The evolution of the lowest energy for all generations shows that the global optimization converged quickly. It is remarkable to find that one potentially stable structures can be readily obtained in the first few generations (labeled by red star in Figure 1A). When refined by DFT, it shows that this structure is the ground state structure of the corresponding combination. In the tests of some other stoichiometric proportions, that is, Mg_3_Al_3_, Mg_2_Al_2_, Mg_1_Al_1_, and Mg_3_Al, the corresponding known structures in the materials project database, that is, mp-1038779, mp-1094987, mp-1038934, and the *L*1_2_ phase (Mendelev et al., 2009), are found by the CALYPSO+DP scheme, which further confirms the validity of our approach.

Next, we use the DP+CALYPSO scheme to construct the convex hull of the Al-Mg system systematically. We restrict the number of atom in the supercell to <32 atoms. In other words, we consider the systems Mg_x_Al_y_{*x* + *y* ≤ 32, *x* ≥ 1, *y* ≥ 1, *x, y* ∈ ℤ}, which represent 496 combinations, or 323 concentrations, in total. According to these parameter settings, the total number of local relaxations is up to 2 × 10^7^. In the following, we consider three prerequisites that should be satisfied for a stable crystal structure: (i) thermodynamic stability, which is estimated by the formation energy and convex hull; (ii) dynamic stability, which can be assessed by phonon dispersions; and (iii) mechanical stability, which is evaluated via elastic constants (Xu et al., 2019).

According to the preliminary tests, we notice that although the DP model can generate energies and forces that are very close to the DFT reference model, small intrinsic error still exists. Therefore, if our goal is to calculate properties with the accuracy of the DFT-based PES landscape, an additional refinement step has to be adopted based on structures selected from a DP+CALYPSO process. Two concepts we pay particular attention to are the formation energy (*E*_*fa*_) and the energy above convex hull (*E*_*abh*_). The formation energy (Haastrup et al., 2018) of an alloy system is the energy required to produce the system from the most stable crystal structures of the individual components, which is defined as

(4) $$\begin{array}{l} E_{fa}=\frac{E\left(\text{M}{\text{g}}_{\text{x}}\text{A}{\text{l}}_{\text{y}}\right)-xE\left(\text{Mg}\right)-yE\left(\text{Al}\right)}{x+y} \end{array}$$

where *E*(Mg_x_Al_y_) is the total energy of the material Mg_x_Al_y_, and *E*(Mg) and *E*(Al) are the average energies of the elements Mg and Al in their stable crystal at 0 K. *E*_*abh*_ measures the energy for a material to decompose into the set of most stable materials with the same chemical composition. A positive *E*_*abh*_ indicates that this material is unstable with respect to such decomposition. A zero *E*_*abh*_ indicates that this is the most stable material at its composition. To accurately determine these properties, we use two criteria for an additional DFT refinement: *E*_*fa*_ being less than 20 meV/atom, and *E*_*abh*_ being <20 meV/atom. We use these two criteria at the same time based on the following reasons. Our goal is to find potential stable or meta-stable structures. Due to the error of the DP model, some structures with positive DP-predicted *E*_*fa*_ may turn negative if we refine it by DFT and vice versa. At the same time, the thermodynamic stability is also controlled by *E*_*abh*_. If *E*_*abh*_ is too high, this structure will decompose into other phases even if this structure has a negative formation energy. In the tested example, we will show that since in general DP exhibits a ~2 meV/atom average error. Compared with DFT, the criteria used here are fairly robust. In contrast, for a previously established empirical model, due to its large intrinsic error, the procedure introduced above is no longer practical, since the number of DFT refinements is so large that little efficiency can be gained.

**Figure 2:**
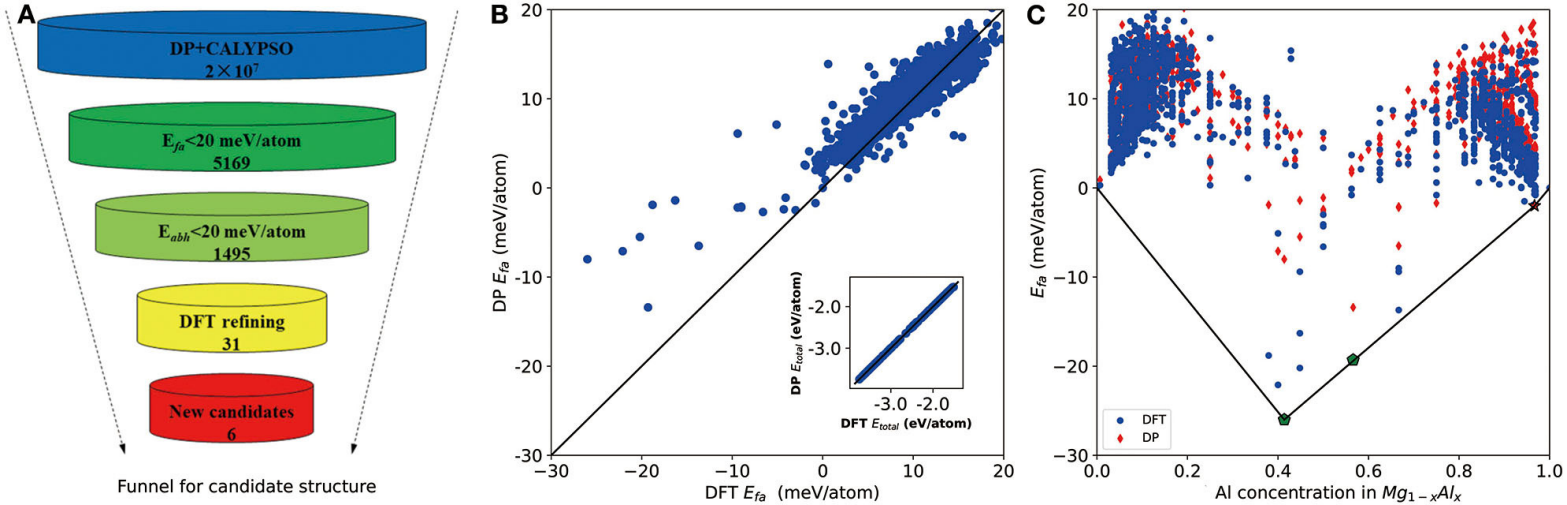
**(A)** The funnel used for screening candidate structures of aluminum–magnesium (Al–Mg), the intermetallic compound via Deep Potential (DP) model. *E*_*fa*_ stands for formation energy and *E*_*abh*_ labels energy above the convex hull. **(B)** Scatter plot of the formation energy calculated by density functional theory (DFT) and DP for potentially candidate structures. The inset shows relationship between average energy calculated by DFT and DP. **(C)** The formation energy as a function of molar fraction of Al atom for different Mg–Al phases where solid line denotes the convex hull constructed by DFT results. The formation energies calculated by DFT are marked by blue circle and DP ones are marked by red diamond. All of known structures from materials project are re-optimized and directly used to construct the convex hull. Pentagon indicates the stable experimental phases and star labels new stable phase.

As shown in Figure 2A, we first use the formation energy *E*_*fa*_ to screen the candidate structures, from which the number of structures is significantly reduced from 2 × 10^7^ to 5,169. Based on these 5,169 DP-optimized structures, a crude convex hull is constructed. Then the criterion for *E*_*abh*_ is introduced to remove the thermodynamically unstable structures, and, finally, 1,495 structures are selected for further DFT refinements. As shown in the inset of Figure 2B, for all DFT refined structures, the energies per atom calculated by DP and DFT are in excellent agreement. The largest and root mean square error (RMSE) of the total energy per atom are about 17 and 2 meV/atom, respectively. As for the formation energy in Figure 2B, the largest error and RMSE are about 15 and 6 meV/atom, respectively. Among them, the errors of the formation energies of experimentally stable phases (Zhuang et al., 2017) Mg_17_Al_12_ and Mg_23_Al_30_ (labeled by pentagon in Figure 2C) are 15 and 6 meV/atom, which confirms the validity of our 20 meV/atom selection criteria.

**Figure 3:**
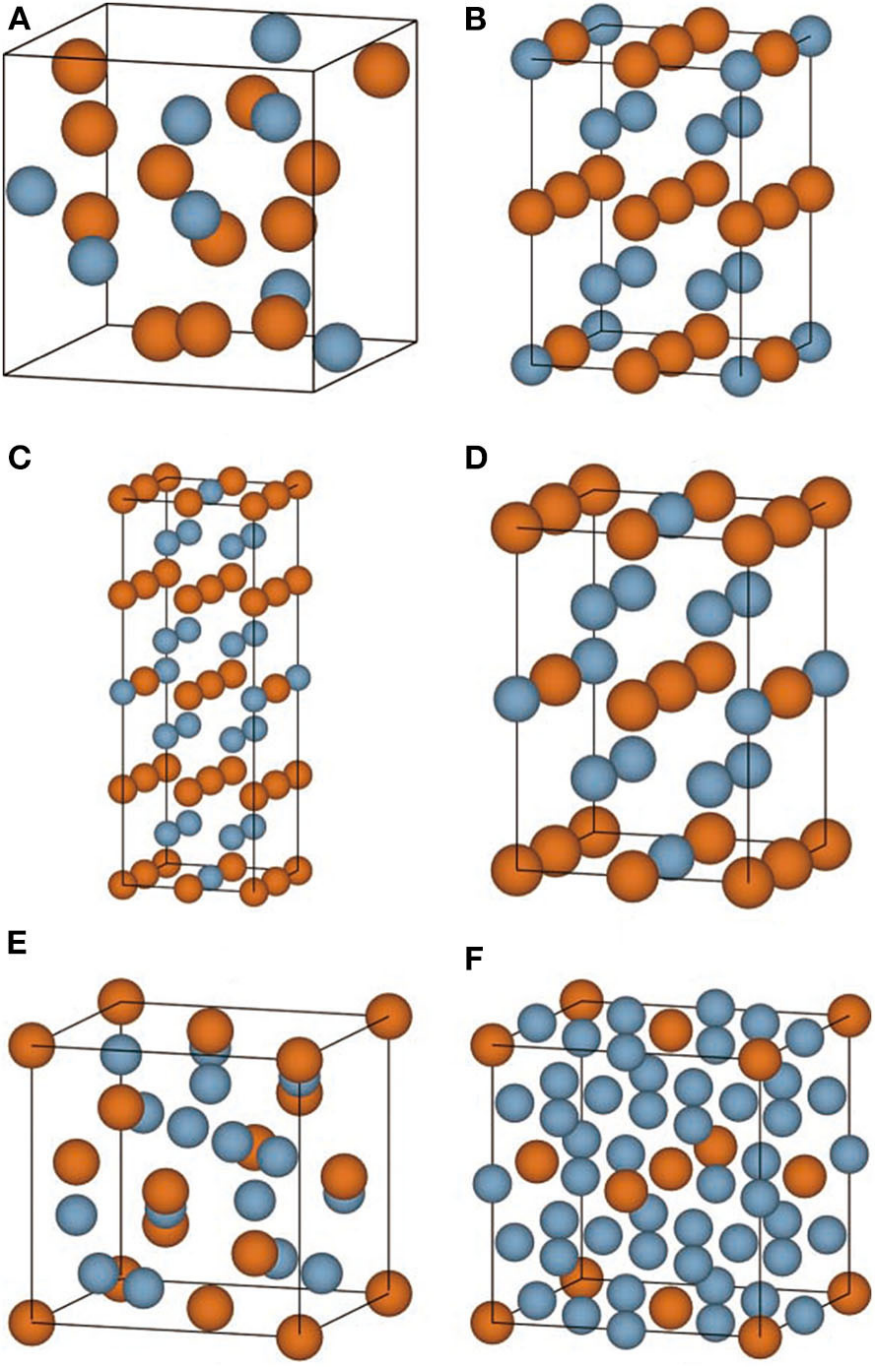
**(A–F)** The side view of conventional crystal structures of Mg_12_Al_8_, Mg_7_Al_9_, Mg_14_Al_18_, Mg_6_Al_10_, Mg_8_Al_16_, and Mg_5_Al_27_ is shown, respectively. The Mg and Al atoms are indicated by yellow and blue ball.

The convex hull based on DFT results is then constructed and presented in Figure 2C, including two experimentally stable structures Mg_17_Al_12_ and Mg_23_Al_30_ labeled by green pentagon. One new stable structure with a formula of MgAl_29_ is discovered and denoted by red star. Based on the DFT-refined convex hull, we look for new structures that are potentially synthesizable by experiments. We use the following criteria: *E*_*abh*_ <20 meV/atom and *E*_*fa*_ <1 meV/atom, where *E*_*abh*_ and *E*_*fa*_ are DFT-calculated values, and obtain 31 potentially candidates, including 1 stable structure and 30 meta-stable structures. However, we may note that most of those newly proposed meta-stable structures locate at the boundary region of the concentration range. That is to say, most of these structures have very low concentration of Al or Mg. Compared with these phases, the phases in the middle region are of more interesting, from which we propose six new meta-stable structures, namely Mg_12_Al_8_, Mg_7_Al_9_, Mg_14_Al_18_, Mg_6_Al_10_, Mg_8_Al_16_, and Mg_5_Al_27_. The corresponding side view of the crystal structures are shown in Figures 3A–F and the geometric structure parameters are listed in Table 1.

**Table 1:** Lattice parameters a(Å), b(Å), c(Å), density ρ (g/cm^3^), space group symbol *S*_*m*_, lattice type, formation energy *E*_*fa*_ (meV/atom), and energy above the convex hull *E*_*abh*_(meV/atom) of Mg_12_Al_8_, Mg_7_Al_9_, Mg_14_Al_18_, Mg_6_Al_10_, Mg_8_Al_16_, and Mg_5_Al_27_ calculated by DFT.

**Formula**	***N*_*atom*_**	**a**	**b**	**c**	**ρ**	***S*_*m*_**	**Lattice type**	***E*_*fa*_**	***E*_*ah*_**
Mg_12_Al_8_	20	7.38	7.38	7.38	2.10	*P*4_332_	Cubic	−22.05	3.09
Mg_7_Al_9_	16	5.98	5.98	8.44	2.27	P4/mmm	Tetragonal	−0.82	18.74
Mg_14_Al_18_	32	5.98	5.98	16.92	2.27	I4/mmm	Tetragonal	−0.10	19.45
Mg_6_Al_10_	16	5.93	5.93	8.43	2.33	I4/mmm	Tetragonal	0.87	17.71
Mg_8_Al_16_	24	7.67	7.67	7.67	2.30	Fd-3m	Cubic	−13.73	1.30
Mg_5_Al_27_	32	8.21	8.21	8.21	2.55	Pm-3m	Cubic	0.60	7.95

As listed in Table 1, the meta-stable structures can be divided into two groups according to their lattice types. Mg_7_Al_9_, Mg_14_Al_18_, and Mg_6_Al_10_ have a tetragonal lattice, whereas Mg_12_Al_8_, Mg_8_Al_16_, and Mg_5_Al_27_ have a cubic lattice. Moreover, all structures have nearly zero or negative formation and small energy above convex hull, which indicates that these structures may be synthesizable by experiments in future.

**Figure 4:**
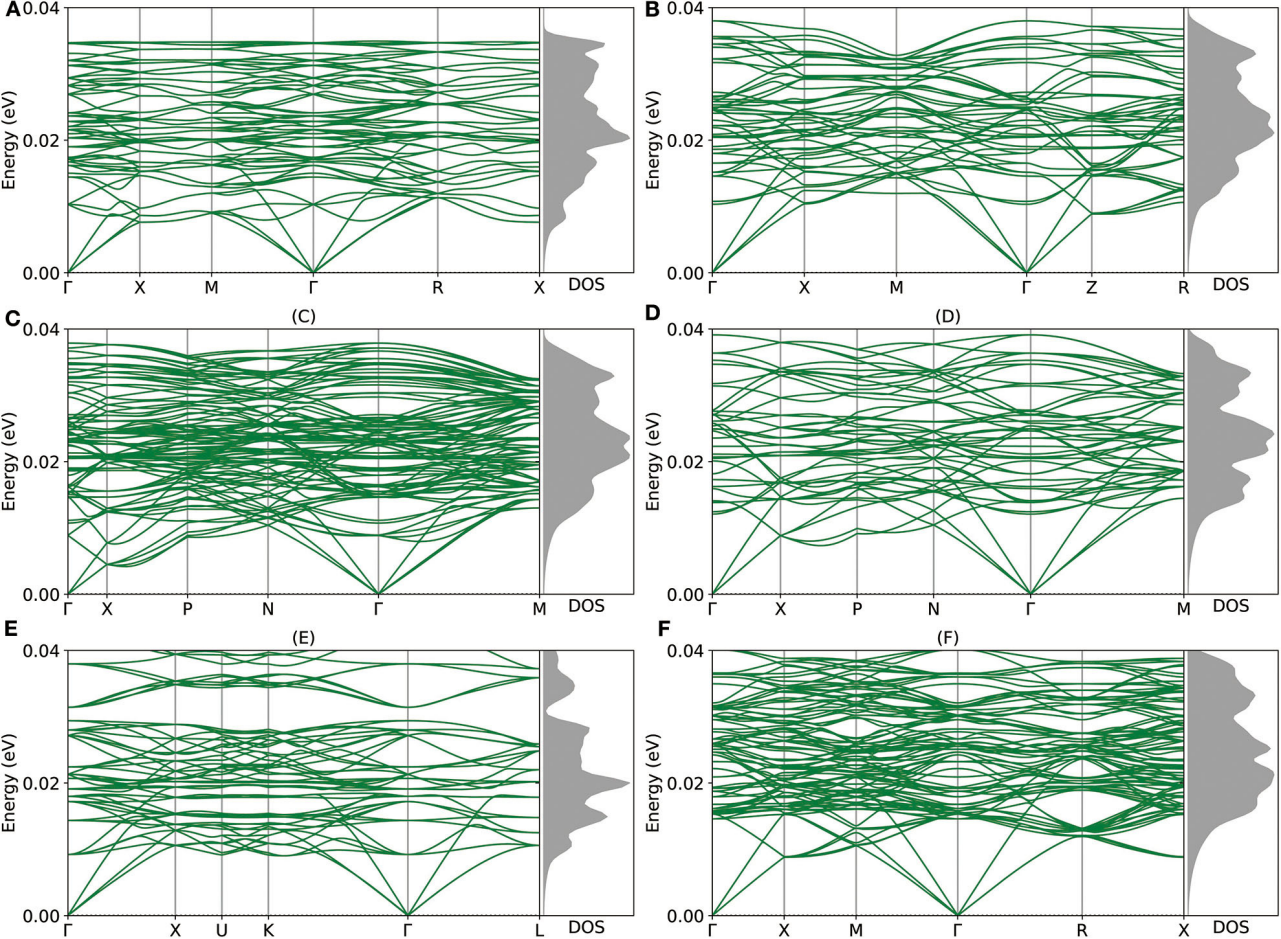
**(A–F)** The phonon structures of Mg_12_Al_8_, Mg_7_Al_9_, Mg_14_Al_18_, Mg_6_Al_10_, Mg_8_Al_16_, and Mg_5_Al_27_ calculated by Deep Potential (DP) model are shown.

Given the encouraging stability metrics above, we proceed to study the dynamic and mechanical stability of these newly proposed intermetallic compounds via DP model. As shown in Figure 4, the phonon structures show no imaginary frequency, which indicates that all of those intermetallic compounds are dynamically stable. As for the mechanical aspect, the elastic stability conditions (Mouhat and Coudert, 2014) for cubic crystals are given as:

(5) $$\begin{array}{l} C_{11}-C_{12}>0,C_{11}+2C_{12}>0,C_{44}>0, \end{array}$$

and those for tetragonal crystals are given as:

(6) $$\begin{array}{l} C_{11}>|C_{12}|,2C_{13}^{2}<C_{33}\left(C_{11}+2C_{12}\right),C_{44}>0,C_{66}>0. \end{array}$$

According to Table 2, both groups of structures meet the elastic stability conditions, which indicates that these 6 intermetallic compounds are mechanically stable. Further, we use the Pugh's ratio *B*_*v*_/*G*_*v*_ to assess the expected average ductility (Pugh, 1954). According to Pugh, a larger *B*_*v*_/*G*_*v*_ value implies a better ductility property. As shown in Table 2, the Pugh's ratio of both Mg_8_Al_16_ and Mg_12_Al_8_ are larger than that of hcp Mg (2.08). In particular, the Pugh's ratio of Mg_12_Al_8_ (2.42) is comparable to that of Al (2.47), so it may have excellent ductility. Considering its lower density (2.10 g/cm^3^) compared with Al (2.72 g/cm^3^), this intermetallic compound may have potential applications in automotive, aerospace, electronic, and device industries if it can be synthesized. In addition, the Mg_5_Al_27_ has a higher Young's modulus among all these structures, which indicates that this material may be applied to manufacture high strength devices.

**Table 2:** Elastic constants, bulk modulus *B*_*v*_ (GPa), shear modulus *G*_*v*_ (GPa), Young's modulus *E*_*v*_ (GPa), Pugh's ratio (*B*_*v*_/*G*_*v*_), and Poisson's ratio ν of Mg_12_Al_8_, Mg_7_Al_9_, Mg_14_Al_18_, Mg_6_Al_10_, Mg_8_Al_16_ and Mg_5_Al_27_ calculated by the DP model.

**Formula**	***C*_11_**	***C*_12_**	***C*_13_**	***C*_33_**	***C*_44_**	***C*_66_**	***B*_*v*_**	***G*_*v*_**	***B*_*v*_/*G*_*v*_**	***E*_*V*_**	**ν**
Mg_9_^*a*^^,^^1^	59.57	26.54	21.05	72.94	15.08	16.12	36.53	17.59	2.08	45.48	0.29
Mg_12_Al_8_	71.26	35.75	36.50	73.15	21.19	21.38	48.14	19.89	2.42	52.45	0.32
Mg_7_Al_9_	94.75	30.55	39.20	84.63	32.91	22.00	53.81	28.08	1.92	71.77	0.28
Mg_14_Al_18_	89.19	31.60	40.27	85.57	32.16	23.48	54.57	27.49	1.99	70.60	0.28
Mg_6_Al_10_	101.77	36.06	42.59	92.14	31.37	23.23	59.65	29.05	2.05	74.97	0.29
Mg_8_Al_16_	97.47	49.80	48.72	95.07	30.68	31.73	65.06	28.35	2.29	74.27	0.31
Mg_5_Al_27_	95.40	39.17	39.87	104.71	37.48	40.21	58.91	35.09	1.68	87.84	0.25
Al_4_^*a*^^,^^2^	117.64	63.34	58.20	108.46	32.55	40.44	78.15	31.65	2.47	83.66	0.32

*The subscript v denotes the Voigt expressions. The same properties of Mg and Al element are also calculated for comparison (here, the minor inconsistency of elastic constants comes from inherent error of DP model and computation error)*.

a *All values are calculated by authors based on DP model*.

1 *Zhuang et al. (2017) give DFT values of C_11_ = 66 GPa, C_12_ =25 GPa, C_13_ = 19 GPa, C_33_ = 70 GPa, C_44_ = 20 GPa, B_v_ = 37 GPa, G_v_ = 21 GPa, B_v_/G_v_ = 1.76 GPa, and E_V_ = 54 GPa*.

2 *Zhang et al. (2019) give DFT values of C_11_ = 111.2 GPa, C_12_ = 61.4 GPa, C_44_ = 36.8 GPa, B_v_ = 78.0 GPa, and G_v_ = 32.1 GPa*.

**Figure 5:**
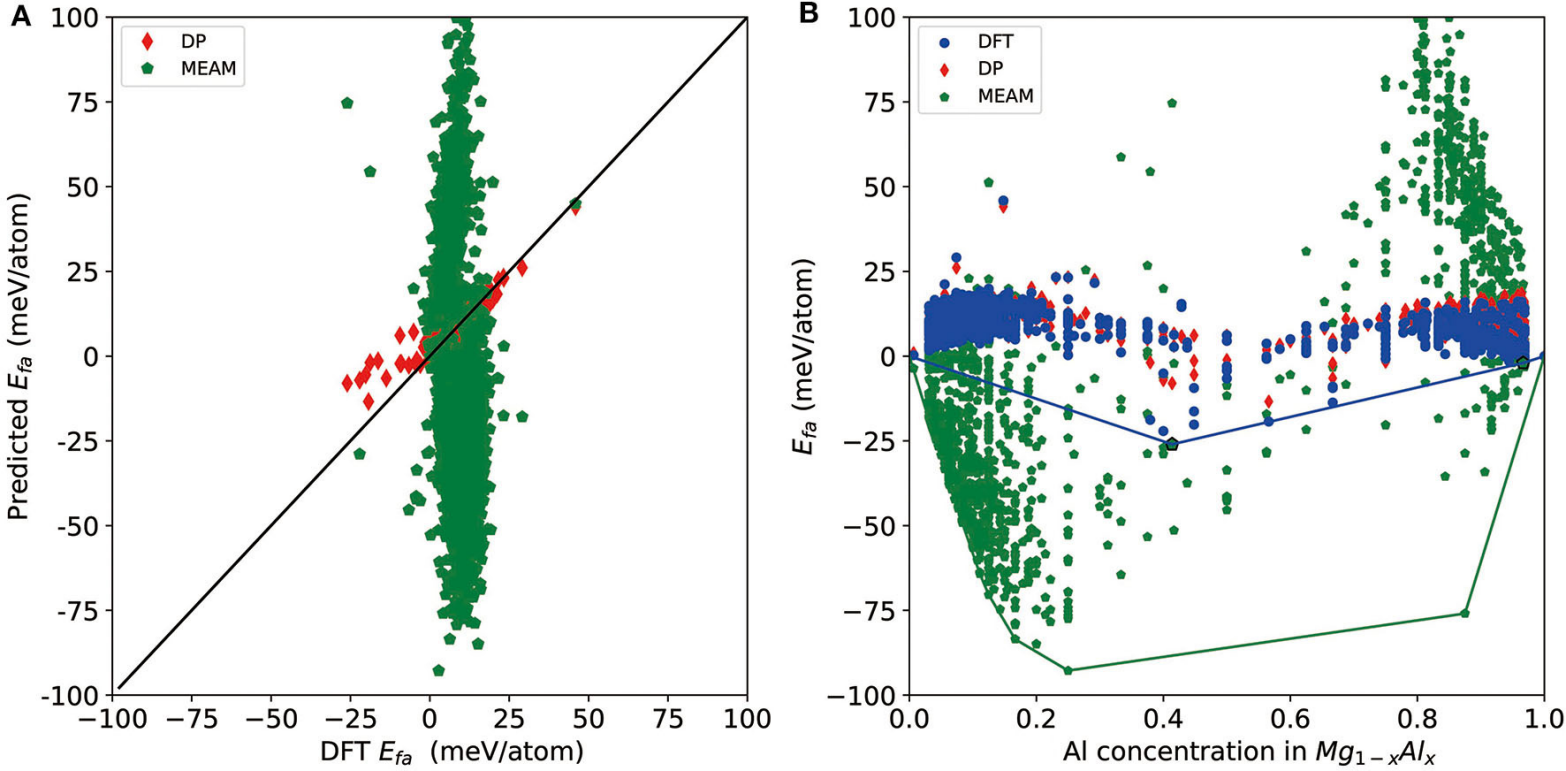
**(A)** Scatter plot of the formation energy calculated by density functional theory (DFT) and Deep Potential (DP) for potentially candidate structures. **(B)** The formation energy as a function of molar fraction of Al atom for different Mg–Al phases where solid line denotes the convex hull constructed by DFT results. The formation energies calculated by DFT, DP, and MEAM are marked by blue circle, red diamond, and green pentagon, respectively.

Finally, we test the accuracy of a recent version of the MEAM potential (Jelinek et al., 2012) and see whether it can be used for a similar task or not. For a direct comparison, we test it on all DFT-refined structures and report the results in Figure 5. As shown by the red diamonds and green pentagons in Figure 5A, MEAM exhibits much larger errors compared with DP for most of the structures, and there are MEAM predictions outside the range of the plot (±100 meV/atom) due to large errors. MEAM results show a large spread on the convex hull plot constructed by DFT results (Figure 5B). For the tested structures, the largest error of *E*_*fa*_ is 204 meV/atom, and the RMSE is 44 meV/atom. Moreover, the largest error of per-atom total energy is 465 meV/atom, and the mean error is 236 meV/atom. As such, if we use MEAM+CALYPSO to do a screening of the structures, the selection criteria for further DFT refinement would be *E*_*fa*_ and *E*_*abh*_ larger than at least 200 meV/atom. As a rough estimation, according to these criteria, ~1 × 10^6^ structures will need to be refined by DFT, which is not computationally feasible at all. Above all, we conclude that although MEAM is very efficient, it cannot be used to improve the efficiency of constructing a convex hull at the level of DFT accuracy.

To demonstrate the efficiency of our DP-based procedure, we use two groups of structures to compare the time performance. One group is composed of several Mg_31_Al structures and the other group consists of MgAl_31_ structures. The test results shows that, compared with DFT relaxation, DP has an average speed-up ratio about 3,700 and 650 for Mg_31_Al and MgAl_31_, respectively, which indicates DP has better time scaling and can be used for larger systems. All tests are performed on Intel(R) Xeon(R) Gold 6248 CPU @ 2.50 GHz.

## Conclusions

In this paper, we demonstrate that the DP+CALYPSO scheme is reliable for crystal structure prediction for binary alloy system in a wide concentration range. As a concrete example, we use this scheme to predict potentially stable intermetallic compounds of the Al–Mg binary system. Six new meta-stable Al–Mg intermetallic compounds are successfully predicted, including Mg_12_Al_8_, Mg_7_Al_9_, Mg_14_Al_18_, Mg_6_Al_10_, Mg_8_Al_16_, and Mg_5_Al_27_. All the meta-stable structures are predicted to have thermodynamic stability, dynamic stability, and mechanical stability. In particular, Mg_12_Al_8_ shows excellent ductility and Mg_5_Al_27_ has high Young's modulus. We remark that the exploration strategy proposed in this work can be combined with the DP-GEN protocol (Zhang et al., 2019, 2020) to generate more training data and improve the DP potential. Moreover, to serve a larger community, DeePMD-kit can be interfaced with other popular general-purpose crystal structure prediction software such as CALYPSO, USPEX, and Pychemia. However, some disadvantages also exits for current scheme, such as expensive cost for training a model, low interface efficiency with CALYPSO, and so on, which limits its application to search complex multicomponent systems with larger number of atoms. We will leave these problems in our future work.

## Data Availability Statement

All datasets presented in this study are included in the article/Supplementary Material.

## Author Contributions

HaiW, HanW, and LZ conceived the idea. HaiW performed the calculations and wrote the script. All authors revised the manuscript.

## Conflict of Interest

The authors declare that the research was conducted in the absence of any commercial or financial relationships that could be construed as a potential conflict of interest.

## Footnotes

^1^ASM https://www.asminternational.org/phase-diagrams

^2^ICSD https://icsd.fiz-karlsruhe.de/search/basic.xhtml

^3^OQMD http://www.oqmd.org

^4^MP https://www.materialsproject.org

^5^deepmd http://www.deepmd.org

^6^See https://github.com/deepmodeling
