# Supplementary material for: Crystal Structure Prediction of Binary Alloys via Deep Potential
Source: Front Chem. 2020 Nov 26;8:589795. doi: 10.3389/fchem.2020.589795 (PMC7732660; doi:10.3389/fchem.2020.589795)
Supplement: Supplementary file 1 [file Data_Sheet_1.PDF]

frontiers

Supplementary Material

1 CRYSTAL STRUCTURES

VASP POSCAR format structure of $Mg_{12}Al_8$, Mg_7Al_9, $Mg_{14}Al_{18}$, Mg_6Al_{10}, Mg_8Al_{16} and Mg_5Al_{27}.

```
Mg12 Al8
1.0
7.381254 0.000000 0.000000
0.000000 7.381254 0.000000
0.000000 0.000000 7.381254
Mg Al
12 8
direct
0.125000 0.703971 0.546029 Mg
0.375000 0.296029 0.046029 Mg
0.875000 0.203971 0.953971 Mg
0.625000 0.796029 0.453971 Mg
0.546029 0.125000 0.703971 Mg
0.046029 0.375000 0.296029 Mg
0.953971 0.875000 0.203971 Mg
0.453971 0.625000 0.796029 Mg
0.703971 0.546029 0.125000 Mg
0.296029 0.046029 0.375000 Mg
0.203971 0.953971 0.875000 Mg
0.796029 0.453971 0.625000 Mg
0.817362 0.817362 0.817362 Al
0.682638 0.182638 0.317362 Al
0.182638 0.317362 0.682638 Al
0.317362 0.682638 0.182638 Al
0.432638 0.432638 0.432638 Al
0.067362 0.567362 0.932638 Al
0.567362 0.932638 0.067362 Al
0.932638 0.067362 0.567362 Al
Mg14 Al18
1.0
5.976662 0.000000 0.000000
0.000000 5.976662 0.000000
0.000000 0.000000 16.915032
Mg Al
14 18
direct
0.000000 0.000000 0.248109 Mg
0.500000 0.000000 0.250000 Mg
0.500000 0.500000 0.251891 Mg
```

```
0.000000 0.500000 0.250000 Mg
0.000000 0.500000 0.000000 Mg
0.500000 0.000000 0.000000 Mg
0.000000 0.000000 0.000000 Mg
0.500000 0.500000 0.748109 Mg
0.000000 0.500000 0.750000 Mg
0.000000 0.000000 0.751891 Mg
0.500000 0.000000 0.750000 Mg
0.500000 0.000000 0.500000 Mg
0.000000 0.500000 0.500000 Mg
0.500000 0.500000 0.500000 Mg
0.254436 0.254436 0.123194 Al
0.745564 0.745564 0.123194 Al
0.745564 0.254436 0.123194 Al
0.254436 0.745564 0.123194 Al
0.245564 0.754436 0.376806 Al
0.754436 0.245564 0.376806 Al
0.245564 0.245564 0.376806 Al
0.754436 0.754436 0.376806 Al
0.500000 0.500000 0.000000 Al
0.754436 0.754436 0.623194 Al
0.245564 0.245564 0.623194 Al
0.245564 0.754436 0.623194 Al
0.754436 0.245564 0.623194 Al
0.745564 0.254436 0.876806 Al
0.254436 0.745564 0.876806 Al
0.745564 0.745564 0.876806 Al
0.254436 0.254436 0.876806 Al
0.000000 0.000000 0.500000 Al
Mg5 Al27
1.0
8.210095 0.000000 0.000000
0.000000 8.210095 0.000000
0.000000 0.000000 8.210095
Mg Al
5 27
direct
0.000000 0.500000 0.500000 Mg
0.500000 0.000000 0.500000 Mg
0.500000 0.500000 0.000000 Mg
0.500000 0.500000 0.500000 Mg
0.000000 0.000000 0.000000 Mg
0.249519 0.750481 0.000000 Al
0.750481 0.249519 0.000000 Al
0.750481 0.750481 0.000000 Al
```

```
0.249519 0.249519 0.000000 Al
0.000000 0.249519 0.750481 Al
0.000000 0.750481 0.249519 Al
0.000000 0.750481 0.750481 Al
0.000000 0.249519 0.249519 Al
0.750481 0.000000 0.249519 Al
0.249519 0.000000 0.750481 Al
0.750481 0.000000 0.750481 Al
0.249519 0.000000 0.249519 Al
0.246817 0.500000 0.246817 Al
0.753183 0.500000 0.246817 Al
0.753183 0.500000 0.753183 Al
0.246817 0.500000 0.753183 Al
0.246817 0.246817 0.500000 Al
0.246817 0.753183 0.500000 Al
0.753183 0.753183 0.500000 Al
0.753183 0.246817 0.500000 Al
0.500000 0.246817 0.246817 Al
0.500000 0.246817 0.753183 Al
0.500000 0.753183 0.753183 Al
0.500000 0.753183 0.246817 Al
0.500000 0.000000 0.000000 Al
0.000000 0.500000 0.000000 Al
0.000000 0.000000 0.500000 Al
Mg6 Al10
1.0
5.926209 0.000000 0.000000
0.000000 5.926209 0.000000
0.000000 0.000000 8.431011
Mg Al
6 10
direct
0.000000 0.500000 0.000000 Mg
0.000000 0.000000 0.000000 Mg
0.500000 0.000000 0.000000 Mg
0.500000 0.000000 0.500000 Mg
0.500000 0.500000 0.500000 Mg
0.000000 0.500000 0.500000 Mg
0.250000 0.750000 0.250000 Al
0.250000 0.250000 0.250000 Al
0.750000 0.250000 0.250000 Al
0.750000 0.750000 0.250000 Al
0.500000 0.500000 0.000000 Al
0.750000 0.250000 0.750000 Al
0.750000 0.750000 0.750000 Al
```

```
0.250000 0.750000 0.750000 Al
0.250000 0.250000 0.750000 Al
0.000000 0.000000 0.500000 Al
Mg7 Al9
1.0
5.982400 0.000000 0.000000
0.000000 5.982400 0.000000
0.000000 0.000000 8.440564
Mg Al
7 9
direct
0.500000 0.000000 0.500000 Mg
0.000000 0.000000 0.500000 Mg
0.000000 0.500000 0.500000 Mg
0.500000 0.500000 0.500000 Mg
0.500000 0.000000 0.000000 Mg
0.000000 0.500000 0.000000 Mg
0.500000 0.500000 0.000000 Mg
0.246675 0.246675 0.754017 Al
0.753325 0.753325 0.754017 Al
0.246675 0.753325 0.245983 Al
0.753325 0.246675 0.245983 Al
0.246675 0.753325 0.754017 Al
0.753325 0.246675 0.754017 Al
0.246675 0.246675 0.245983 Al
0.753325 0.753325 0.245983 Al
0.000000 0.000000 0.000000 Al
Mg8 Al16
1.0
7.672734 0.000000 0.000000
0.000000 7.672734 0.000000
0.000000 0.000000 7.672734
Mg Al
8 16
direct
0.250000 0.750000 0.750000 Mg
0.000000 0.000000 0.000000 Mg
0.250000 0.250000 0.250000 Mg
0.000000 0.500000 0.500000 Mg
0.750000 0.750000 0.250000 Mg
0.500000 0.000000 0.500000 Mg
0.750000 0.250000 0.750000 Mg
0.500000 0.500000 0.000000 Mg
0.875000 0.875000 0.625000 Al
0.625000 0.875000 0.875000 Al
```

```
0.125000 0.125000 0.625000 Al
0.375000 0.125000 0.875000 Al
0.875000 0.375000 0.125000 Al
0.625000 0.375000 0.375000 Al
0.125000 0.625000 0.125000 Al
0.375000 0.625000 0.375000 Al
0.375000 0.875000 0.125000 Al
0.125000 0.875000 0.375000 Al
0.625000 0.125000 0.125000 Al
0.875000 0.125000 0.375000 Al
0.375000 0.375000 0.625000 Al
0.125000 0.375000 0.875000 Al
0.625000 0.625000 0.625000 Al
0.875000 0.625000 0.875000 Al
```
